# Spatial working memory deficits in GluA1 AMPA receptor subunit knockout mice reflect impaired short-term habituation: Evidence for Wagner's dual-process memory model

**DOI:** 10.1016/j.neuropsychologia.2010.03.018

**Published:** 2010-07

**Authors:** David J. Sanderson, Stephen B. McHugh, Mark A. Good, Rolf Sprengel, Peter H. Seeburg, J. Nicholas P. Rawlins, David M. Bannerman

**Affiliations:** a Department of Experimental Psychology, University of Oxford, South Parks Road, Oxford, OX1 3UD, UK; b School of Psychology, Cardiff University, Tower Building, Park Place, Cardiff, CF10 3AT, UK; c Max-Planck Institute of Medical Research, Department of Molecular Neurobiology, D-69120 Heidelberg, Jahnstrasse 29, Germany

**Keywords:** Spatial learning, Recognition memory, AMPA receptors, Synaptic plasticity, Hippocampus, Lesion

## Abstract

Genetically modified mice, lacking the GluA1 AMPA receptor subunit, are impaired on spatial working memory tasks, but display normal acquisition of spatial reference memory tasks. One explanation for this dissociation is that working memory, win-shift performance engages a GluA1-dependent, non-associative, short-term memory process through which animals choose relatively novel arms in preference to relatively familiar options. In contrast, spatial reference memory, as exemplified by the Morris water maze task, reflects a GluA1-independent, associative, long-term memory mechanism. These results can be accommodated by Wagner's dual-process model of memory in which short and long-term memory mechanisms exist in parallel and, under certain circumstances, compete with each other. According to our analysis, *GluA1*^−/−^ mice lack short-term memory for recently experienced spatial stimuli. One consequence of this impairment is that these stimuli should remain surprising and thus be better able to form long-term associative representations. Consistent with this hypothesis, we have recently shown that long-term spatial memory for recently visited locations is enhanced in *GluA1*^−/−^ mice, despite impairments in hippocampal synaptic plasticity. Taken together, these results support a role for GluA1-containing AMPA receptors in short-term habituation, and in modulating the intensity or perceived salience of stimuli.

## 1 Introduction

The ability to learn and remember spatial locations, and to associate them with other stimuli, is essential for adaptive behaviour in many species. Whereas some spatial tasks can be solved purely on the basis of *egocentric* (self-centred) information (e.g. vestibular or proprioceptive cues) that will change every time the animal moves, other spatial tasks require encoding the relationship between salient features of the environment to create an *allocentric* (other-centred) representation that is independent of the animal's current location. Such a representation has been termed a cognitive map (O’Keefe & Nadel, 1978).

There is considerable evidence from a variety of sources that the hippocampus in humans and other animals plays an important role in allocentric spatial memory. Patients with hippocampal pathology, whether as a result of surgery, ischaemic brain damage or as a consequence of clinical conditions such as Alzheimer's disease, have problems with spatial orientation, and experience the feeling of being “lost” even in surroundings with which they have considerable experience (e.g. de Ipolyi, Rankin, Mucke, Miller, & Gorno-Tempini, 2007). Moreover, normal, healthy individuals exhibit activation of brain areas in the hippocampal formation during functional MRI studies when performing tasks in which they are required to navigate around virtual environments (e.g. Maguire, Burgess, & O’Keefe, 1999). Spatial information is likely to provide a very important contextual cue for retrieving other memories, and thus it has been widely suggested to be a key component of human episodic memory (O’Keefe & Nadel, 1978, p. 381).

In rodents, evidence for a hippocampal role in spatial learning and memory has come from two main sources. First, cells in the hippocampi of behaving rats have been found that selectively increased their firing rate only when the rat occupied a well-defined region of the environment, the ‘place field’, and rarely fired outside the place field (O’Keefe & Dostrovsky, 1971). Logically, these cells were named ‘place cells,’ although it is important to note that the firing rate of hippocampal neurons has also been shown, on other occasions, to correlate as reliably with non-spatial stimuli in other experimental situations (e.g. Wood, Dudchenko, & Eichenbaum, 1999; Wood, Dudchenko, Robitsek, & Eichenbaum, 2000). O’Keefe and Nadel (1978) suggested that these place cells provided the neural substrate for a cognitive map.

The second major source of evidence comes from lesion studies. Damage to the hippocampal system impairs allocentric (Morris, Garrud, Rawlins, & O’Keefe, 1982), but not simple egocentric spatial memory tasks in rodents (de Bruin, Moita, de Brabander, & Joosten, 2001; Oliveira, Bueno, Pomarico, & Gugliano, 1997; Packard & McGaugh, 1996; Warburton, Baird, & Aggleton, 1997). There are numerous reports in the literature detailing effects of hippocampal lesions on memory tasks that, at least intuitively, are deemed to have a spatial component, some of which are described later in this review. Studies of lesioned animals can inform as to whether a brain region is necessary for a particular task but, as with human neuroimaging studies, they offer little insight into the molecular and synaptic mechanisms that underlie memory function.

In order to target the synaptic mechanisms of memory, other, subtler, experimental interventions are required. Importantly, experiments using genetically modified mice, in which specific neurobiological pathways can be manipulated, have revealed dissociations between different types of spatial memory that were not evident on the basis of lesion experiments or from imaging studies. In particular, studies with mutant mice have revealed the presence of two dissociable spatial information processing mechanisms. In this review we will describe experiments from studies with mice possessing a GluA1 AMPA receptor subunit knockout and illustrate how the behavioural profile of these mice can be accommodated by a long-standing model of animal learning proposed by Wagner (1981).

## 2 AMPA receptors and hippocampus-dependent spatial memory

AMPA receptors mediate fast synaptic transmission between neurons in the brain. It is not surprising, therefore, that acquisition of the standard, fixed location, hidden escape platform, spatial reference memory version of the watermaze task is impaired when an AMPA receptor antagonist (LY326325) is infused directly into the hippocampus (Riedel et al., 1999). The expression of previously acquired spatial information was also disrupted by intra-hippocampal AMPA receptor blockade. Animals that had been trained on the watermaze task after vehicle infusions then received a memory test, but now following infusion of the AMPA receptor antagonist. Their memory performance was now impaired, confirming a role for hippocampal AMPA receptors during spatial information processing.

The AMPA subtype of excitatory amino acid glutamate receptor is a hetero-oligomeric protein complex consisting of combinations of four different kinds of subunits (GluA1–GluA4; GluR-A–GluR-D; GluR1–GluR4), each encoded by a separate gene (gria 1–4, Wisden & Seeburg, 1993). Investigating the contribution that these different subunits make to spatial memory performance was initially hindered by the lack of subunit-selective ligands. However, the development of genetically modified mice in which the different AMPA receptor subunits can be selectively deleted or modified has now allowed their individual contributions to be studied. For example, genetically modified mice lacking the GluA2 (GluR-B) AMPA receptor subunit from principal neurons in the post-natal forebrain (GluA2^ΔFb^ mice) exhibit a reduction in excitatory synaptic transmission and deficits on a number of spatial memory tasks (Shimshek et al., 2006). They were impaired at learning which arm of a Y-maze (defined by the allocentric spatial cues) always contained a sweet milk reward, and were also impaired on a T-maze spatial non-matching-to-place (rewarded alternation) task. In contrast to GluA2^ΔFb^ mice, GluA1 AMPA receptor subunit knockout (GluA1^−/−^) mice exhibit a very different behavioural phenotype, demonstrating a clear dissociation between two different kinds of spatial memory (spatial reference memory and spatial working memory). Before describing the behavioural phenotype of these mice, it is worth first describing the biochemical and electrophysiological consequences of GluA1 knockout.

## 3 GluA1 knockout mice

The GluA1^−/−^ mouse is a constitutive knockout in which functional GluA1 genes (gria 1) are absent in all cells, including those in the brain, throughout the entire lifetime of the animal (Zamanillo et al., 1999). The expression levels of other glutamate receptor subunits (e.g. GluA2, GluA3, GluN1, GluN2A), however, remain unchanged. GluA1^−/−^ mice exhibit normal development, life expectancy and fine structure of neuronal dendrites and synapses. They do, however, exhibit a marked reduction in the number of functional AMPA receptors. What AMPA receptors are available are preferentially targeted to synapses. Thus, soma-patch currents recorded in CA1 pyramidal cells are strongly reduced. In the original description of these mice, it was reported that glutamatergic synaptic currents were largely unaltered (Zamanillo et al., 1999), although in subsequent studies it was shown that, in fact, the AMPA receptor-mediated synaptic currents were also reduced in GluA1^−/−^ mice (Andrasfalvy, Smith, Borchardt, Sprengel, & Magee, 2003; Jensen et al., 2003). For example, the mean CA1 field EPSP in GluA1^−/−^ mice, in response to a single test pulse of 70 μA, was only 65% of the mean field EPSP in wild-type mice (Romberg et al., 2009). Thus, excitatory synaptic transmission is attenuated in these animals.

Deletion of GluA1 also affects hippocampal synaptic plasticity. Although the induction of hippocampal long-term potentiation (LTP), the most commonly studied form of hippocampal synaptic plasticity, requires the NMDA subtype of glutamate receptor (Collingridge, Kehl, & McLennan, 1983), the continued expression of LTP depends, at least in part, on the translocation of additional AMPA receptors into the post-synaptic membrane (see Kessels & Malinow, 2009 for reviews; Malinow & Malenka, 2002). In turn, this activity-dependent insertion of AMPA receptors may depend, in part, on the GluA1 subunit (Shi, Hayashi, Esteban, & Malinow, 2001). Although the mechanism underlying the delivery and insertion of GluA1-containing AMPA receptors into the post-synaptic membrane is not fully understood, it is thought to involve the phosphorylation of key amino acid residues on the GluA1 subunit.

Consistent with this putative role for GluA1-containing AMPA receptors in the strengthening of synaptic connections, initial electrophysiological characterization of *GluA1*^−/−^ mice showed that hippocampal long-term potentiation (LTP), induced by high frequency tetanic stimulation, was abolished at Schaffer collateral – CA1 pyramidal cell synapses during *in vitro* recordings made in slice preparations from adult animals (Zamanillo et al., 1999). However, other studies have revealed different results. For example, Frey, Sprengel, and Nevian (2009) recently showed that spike-timing dependent plasticity in CA1 pyramidal cells is GluA1-independent. Furthermore, other studies, using a theta-burst induction paradigm, revealed a gradually developing form of LTP in the knockouts (Hoffman, Sprengel, & Sakmann, 2002; Romberg et al., 2009). In these studies, the amount of LTP in the *GluA1*^−/−^ mice was found to be indistinguishable from that in the wild-types, 20–45 min after theta-burst induction (but see also Erickson, Maramara, & Lisman, 2009). Thus, following theta-burst stimulation, at least, GluA1 appears to contribute more to the early, rapidly decaying component of LTP, which might actually be considered more akin to a form of short-term potentiation (STP). Consistent with these findings, Erickson et al. (2009) have recently shown that weaker stimuli, which are insufficient to induce LTP, result in STP in wild-type mice, and that this is greatly reduced in the *GluA1*^−/−^ mice.

## 4 Spatial reference and working memory – GluA1 knockout mice

**Fig. 1:**
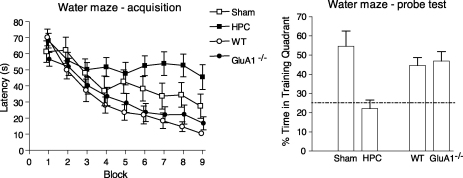
Hippocampal lesions, but not *GluA1* deletion impair performance of spatial reference memory in the water maze task. Water maze–acquisition: mean escape latency (±S.E.M.) to find the platform across nine days of training for sham (Sham) and hippocampal lesioned mice (HPC), and for wild-type (WT) and *GluA1*^−/−^ mice. Water maze–probe test: mean percent time in the training quadrant (±S.E.M.) during a probe trial in which the platform is removed from the pool and the mice are allowed to swim freely. The dashed line indicates chance performance. Sham and hippocampal lesioned mice results reproduced from Deacon et al. (2002). Wild-type and *GluA1*^−/−^ mice results reproduced from Reisel et al. (2002).

Whatever the impairments in hippocampal synaptic plasticity, *GluA1*^−/−^ mice display normal spatial memory on the hidden platform Morris water maze task (Reisel et al., 2002; Zamanillo et al., 1999). In this task, rodents are trained to find a hidden escape platform that remains in a fixed spatial location on every trial. The rat is released into the water from various different starting points around the maze and is required to use the extramaze cues located around the testing room to navigate towards the location of the escape platform. This paradigm is often referred to as a spatial reference memory task. Learning is dependent on the formation of associations between extramaze cues and the escape platform, a relationship that remains consistent across all training trials. Rats or mice with hippocampal lesions take more time and travel greater distances to find the platform compared to controls (Deacon, Bannerman, Kirby, Croucher, & Rawlins, 2002; Morris et al., 1982; Morris, Schenk, Tweedie, & Jarrard, 1990). After training, a probe (or transfer) test can be conducted, in which the platform is removed from the pool and a measure of the animal's preference for searching in the platform location is obtained. Control animals typically spend most of their time searching in the quadrant where the platform was previously located. In contrast, hippocampal lesioned animals display no such preference and exhibit chance levels of performance. However, in marked contrast to animals with hippocampal lesions, *GluA1*^−/−^ mice are indistinguishable from wild-type littermate controls in acquiring this task, both in terms of path lengths during training and probe test performance (see Fig. 1). It is also worth noting that the phenotype of the *GluA1*^−/−^ mice on this task is very different from the consequences of blocking all fast synaptic transmission in the hippocampus with an AMPA receptor antagonist (e.g. LY326325, Riedel et al., 1999), thus demonstrating an important difference between losing just the GluA1 subunit and losing all AMPA receptor-mediated transmission.

Despite normal acquisition and retention of spatial reference memory tasks, *GluA1*^−/−^ mice are dramatically impaired on a different kind of spatial memory test. Rewarded alternation (or non-matching to position) on the elevated T-maze is often described as a spatial working memory task. Correct performance is reliant on the ability to remember unique information from a single trial, and to choose the correct spatial response (from alternatives that are variably correct or incorrect), based on this trial-specific information. Spatial working memory performance is also extremely sensitive to septo-hippocampal lesions, both in rats (Rawlins & Olton, 1982), and mice (Deacon et al., 2002). During the task, mice receive a sample trial in which they are forced to enter either the left or right goal arm where they receive a food reward. Immediately after the forced sample trial, mice then receive a free choice trial in which they are rewarded for entering the arm that was not previously visited. They are not rewarded if they re-visit the arm that was entered on the sample trial. In other words they are rewarded for alternating or adopting win-shift behaviour. *GluA1*^−/−^ mice exhibit a robust and reliable impairment on the T-maze rewarded alternation task. Indeed, they never attain levels of performance that are above chance (i.e. 50% correct), even after extended training (Reisel et al., 2002). Thus, on spatial working memory tasks *GluA1*^−/−^ mice *do* resemble animals with hippocampal lesions.

## 5 Spatial reference and working memory – Olton's terminology

**Fig. 2:**
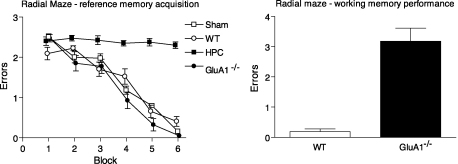
Hippocampal lesions, but not *GluA1* knockout impair acquisition of the spatial reference memory component of the radial maze task. Radial-arm maze–reference memory acquisition: mean number of reference memory errors per trial (±S.E.M.) during 6 blocks of training (4 trials per block). Mice were trained to discriminate between 3 baited arms and 3 non-baited arms of a 6 arm radial maze. Doors prevented mice re-entering arms they had already visited on that particular visit to the maze (i.e. prevented working memory errors) during this stage of the experiment. Sham lesioned (Sham), wild-type (WT), and *Glua1*^−/−^ mice all acquired the task at a similar rate. Hippocampal lesioned mice (HPC) were completely unable to learn which arms were baited and which arms were not baited. Radial-arm maze–working memory performance: mean number of working memory errors per trial (±S.E.M.) for wild-type (WT) and *Glua1*^−/−^ mice. During this stage of testing mice were still rewarded in the same 3 arms of the maze and not rewarded in the 3 non-baited arms, but now they were allowed to re-enter arms as often as they liked, and rewards were not replaced within a trial. Data reproduced from Schmitt et al. (2003).

*GluA1*^−/−^ mice perform normally on the spatial reference memory water maze task, but are impaired on the T-maze spatial working memory task. In the present context, we use the term working memory to refer to memory in which trial-specific information is used to select between response options that are variably correct or incorrect. This particular idea of distinct working memory and reference memory systems was first proposed by Honig (1978), and later elaborated upon by Olton, Becker, and Handlemann (1979). The distinction between spatial working and reference memory is perhaps best illustrated in the context of Olton's radial maze task. The radial maze consists of a number of arms (often 6, 8 or 12) that radiate out from a central platform like spokes on a bicycle wheel. There are a variety of different ways that the radial maze can be used to study different aspects of spatial memory. We adopted a version of the task in which 3 out of 6 arms were baited and the remaining 3 arms were never baited. The animal must first learn to discriminate between the baited and never-baited arms. This is what Olton called the spatial reference memory component of the task and if an animal enters an arm that is never baited then this is scored as a reference memory error. As with the reference memory version of the water maze task, hippocampal lesioned mice are impaired at acquiring the reference memory component of the radial-arm maze task and never learn to discriminate between the baited and never-baited arms (Schmitt, Deacon, Seeburg, Rawlins, & Bannerman, 2003). Again, in parallel with the water maze findings, however, the *GluA1*^−/−^ mice are perfectly capable of learning to discriminate between the baited and never-baited arms, and do so as efficiently as wild-type controls (see Fig. 2).

If the food rewards are not replaced within a trial, then the animal needs to keep track of which arms they have already visited on that particular trial for efficient performance (i.e. they must adopt a win-shift behavioural response). If an animal enters an arm that is normally baited, but which it has already entered on that trial (and so now is not rewarded), then this is scored as a working memory error. As with the T-maze rewarded alternation task (Reisel et al., 2002), *GluA1*^−/−^ mice exhibit a pronounced spatial working memory deficit and fail to exhibit the appropriate win-shift behaviour on the radial maze (Schmitt et al., 2003, see Fig. 2).

## 6 The GluA1 conundrum

Thus, data from *GluA1*^−/−^ mice suggest there are two dissociable forms of spatial information processing, both of which depend on the hippocampus for their expression. There is a rapid, GluA1-dependent form of information processing which underlies, or at least contributes to, spatial working memory performance on tasks like the T-maze and the working memory component of the radial-arm maze. Then there is also a gradually acquired or incrementally strengthened spatial reference memory mechanism that is GluA1-independent. Olton's working/reference memory terminology is of limited value in terms of understanding the psychological processes that might underlie performance on these different behavioural tasks The terms working and reference memory provide a useful description of the tasks themselves, but little more. Furthermore, in terms of understanding the psychological processes that might underlie performance on the different tasks, the effects of *GluA1* deletion on different spatial memory tasks provides something of a conundrum. Strikingly it would appear that whatever synaptic mechanism underlies spatial working memory performance, it plays no part in spatial reference memory acquisition, a result that needs to be incorporated into any model of hippocampal function.

So why does the absence of spatial working memory not hinder performance of *GluA1*^−/−^ mice on spatial reference memory tasks like the standard water maze paradigm? Or put another way, why does the presence of a spatial working memory mechanism not give wild-type mice an advantage over *GluA1*^−/−^ mice? When *GluA1*^−/−^ mice were tested on the T-maze rewarded alternation paradigm they resembled mice with hippocampal lesions. Indeed, it is worth pointing out that the *GluA1*^−/−^ mice performed every bit as badly as hippocampal lesioned mice. However, at the same time, they performed every bit as well as controls on the hippocampus-dependent reference memory water maze task, both in terms of escape latencies and path lengths during acquisition, and in terms of probe test performance. It seems unlikely, therefore, that the differential outcomes reflect the fact that working memory tasks are simply more sensitive than reference memory tasks to hippocampal dysfunction.

In terms of psychological processes, it has been suggested that win-shift performance on spatial working memory tasks like the T-maze or radial maze may be reliant on a one-trial, episodic, or episodic-like, memory system (e.g. Aggleton & Pearce, 2001; Montgomery & Buzsaki, 2007; Morris, 2006; Pastalkova, Itskov, Amarasingham, & Buzsaki, 2008). Indeed, we previously raised the possibility that the spatial working memory performance deficits in *GluA1*^−/−^ mice could reflect an episodic-like memory impairment in these animals (Schmitt et al., 2004, 2003, 2005). However, if this is the case, then why does a memory of the episode that constitutes the previous trial (trial *n* − 1) not help wild-type animals on the present trial (trial *n*) during reference memory acquisition of the water maze task, for example? The data from *GluA1*^−/−^ mice, which exhibit chance performance on win-shift maze tasks, but normal reference memory acquisition, suggest that it is of no benefit. Thus, either (i) an episodic memory account of the spatial working memory deficit in *GluA1*^−/−^ mice is wrong, or (ii) episodic-like memory is not important for the formation of long-term spatial memories on reference memory tasks, or (iii) both of these assumptions are wrong. So how do we explain the relationship between these GluA1-dependent and independent spatial information processing mechanisms?

## 7 An alternative account – GluA1 and short-term habituation

It is generally considered that spatial reference memory acquisition, whether on aversively motivated tasks like the Morris water maze, or using appetitively motivated paradigms such as the radial-arm maze, involves forming associations between spatial locations and outcomes, such as the presence of an escape platform or a food reward, which are stored in long-term memory. It is not unreasonable to think that this might require the strengthening of synaptic connections between the neurons that represent the particular spatial location and the neurons that represent the reward, possibly through an LTP-like mechanism. But what are the likely psychological and neurobiological processes that might underlie spatial working memory performance?

It is interesting to note that in normal animals, performance on win-shift maze tasks, such as T-maze rewarded alternation, is very often well above chance levels from the beginning of training, and indeed in some instances animals can exhibit greater than 90% choice accuracy from the very start of testing (e.g. Bannerman et al., 1999). This reflects the fact that animals will alternate spontaneously, even in the absence of any food rewards. Indeed, discrete-trial spontaneous alternation is a widely used test of spatial memory in its own right. In this task, the animal is allowed to enter one of the goal arms of a T-maze during a sample trial and explore for a short period of time (e.g. for 30 s). It is then removed from the maze and, after a short delay (e.g. 15 s), it is returned to the maze and given a free choice of either arm. Animals are given a number of these trials and the number of alternations is calculated. Normal mice show a strong preference to alternate, even in the absence of any food rewards in the arms. Spontaneous alternation performance is sensitive to both hippocampal lesions (Deacon et al., 2002) and also to *GluA1* deletion (Bannerman et al., 2004). Thus, although it may appear that the solution to spatial working memory tasks, like T-maze rewarded alternation or the win-shift paradigm on the radial maze, requires the ability to learn the non-matching rule, this may not be the case. Instead, successful performance may, at least to a considerable extent, reflect an innate preference to alternate, a preference that does not need to be acquired. It is of course true that in some experiments performance levels do start closer to chance levels and increase with training, but this may simply reflect an increase in the ability to discriminate between different spatial locations (e.g. a perceptual learning effect, Trobalon, Sansa, Chamizo, & Mackintosh, 1991), rather than the acquisition of any rule.

The spontaneous preference to alternate that normal animals exhibit reflects novelty-seeking behaviour. This behavioural phenomenon is caused by habituation to the cues defining the familiar arm, which in turn renders the cues less novel and thus less interesting. Habituation is defined as the decline in the tendency to respond to a stimulus following previous exposure. Thus, the process of habituation may contribute to performance on win-shift tasks in which animals are required to choose the more novel option in preference to the more familiar option for reward. For example, as a consequence of experiencing a particular spatial location (e.g. the goal arm visited during the sample run), there will be a subsequent, short-term reduction in the tendency to explore that same location relative to the other arms available. Thus, animals will show an increased tendency to explore the relatively less familiar or relatively more novel option(s). Thus, the performance of normal animals on win-shift procedures such as the T-maze and radial maze tasks may reflect, at least in part, habituation processes as opposed to the acquisition of a specific non-matching rule. Our working premise, therefore, is that stimulus-specific, short-term habituation processes may support spatial working memory performance on win-shift maze tasks in rodents. Theoretical accounts of the mechanisms underlying habituation may therefore provide a suitable framework for understanding the behavioural dissociations presented by *GluA1*^−/−^ mice. We shall return to this in Section 9.

## 8 GluA1 and spatial recognition memory

The reduction in exploration of an environment, as measured by a reduction in locomotor activity, during continuous exposure could reflect the habituation to that environment. However, it could also reflect non-specific changes in activity levels such as reduced arousal or increased fatigue. To demonstrate that habituation is stimulus-specific rather than simply a non-specific, general decline in responding, it is necessary to show that responding can be restored by a novel stimulus. This can be achieved by presenting animals with a simultaneous choice between a familiar, habituated spatial location and an unvisited, novel location. Habituation will result in a decline in the ability of the familiar spatial location to elicit exploratory behaviour and thus animals will show preferential exploration of the novel over the familiar place. We have recently assessed spatial novelty preference in *GluA1*^−/−^ mice using a simple Y-maze procedure (Sanderson et al., 2007). The design of this test is very similar to the widely used test of object recognition memory in rodents (Ennaceur & Delacour, 1988). During object recognition tests, rodents are allowed to explore a sample object (object A^1^) before then being presented with a choice between a duplicate of this familiar object (A^2^) and a novel object (B). Normal animals show a preference for exploring (contacting and sampling) the novel object B as a result of previous exposure (or habituation) to object A.

**Fig. 3:**
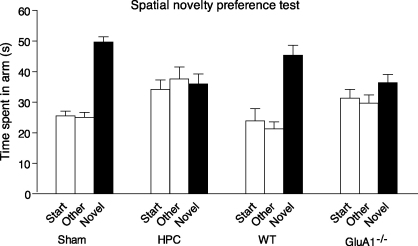
Hippocampal lesions and *GluA1* knockout both impair performance on a spatial novelty preference test. Mean time spent in arms (±S.E.M.). Sham and wild-type (WT) mice exhibit a preference for a previously unexposed (Novel) arm of a Y-maze over two familiar arms to which they have previously been exposed (Start and Sample). *GluA1* knockout mice (*GluA1*^−/−^) and hippocampal lesioned mice (HPC) did not show a significant preference for the novel arm. Data reproduced from Sanderson et al. (2007).

In the spatial novelty preference test, mice were first exposed to two arms of a Y-maze (the Start and Sample arms) for 5 min. Access to the third arm was blocked during this period. The mouse was removed from the maze and, after a short, 1-min, delay (spent in a holding cage), it was then returned to the maze and allowed a free choice to explore all three arms. Normal mice showed a preference for the previously unvisited, novel arm over the two familiar (Start and Sample) arms. In contrast, mice with hippocampal lesions failed to show a preference for the novel arm during the test phase (Sanderson et al., 2007), consistent with an impaired ability to discriminate between the arms on the basis of their spatial location (O’Keefe & Nadel, 1978). Importantly, recent work from our laboratory has also shown that this short-term spatial novelty preference is dependent on GluA1. Similar to hippocampal lesioned animals, *GluA1*^−/−^ mice failed to show any preference for the novel arm when tested after a 1 min interval (Sanderson et al., 2007, see Fig. 3). This is in marked contrast to the ability of *GluA1*^−/−^ mice to discriminate between rewarded and non-rewarded spatial locations in spatial reference memory tasks (e.g. on the radial-arm maze or in the Morris water maze task). Therefore, GluA1 is necessary for short-term spatial habituation, but not for associative, long-term spatial memory.

## 9 Wagner's SOP theory; short and long-term habituation

Wagner (1976, 1981) suggested that the memory processes that underlie short-term habituation are qualitatively different from the processes that result in more durable, long-term memory. These separate short-term and long-term memory processes can occur independently of one another. Thus, this theory may provide an account of the dissociation of spatial working and reference memory in *GluA1*^−/−^ mice. In the following section Wagner's sometimes opponent process (SOP) theory (Wagner, 1981) and its account of short-term and long-term habituation are described.

**Fig. 4:**
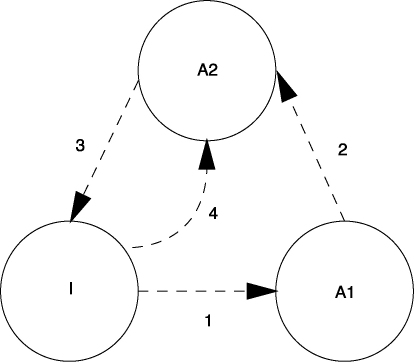
The states of activation, which elements of a memory can reside, and the permissible transitional routes between states, according to Wagner (1981). Presentation of a stimulus leads to a proportion of its elements transferring from the inactive state (I) to the A1 state (route 1). Elements then decay to a secondary activation state, A2 (route 2), before returning to an inactive state, I (route 3). Elements that are active in the A2 state cannot return to the A1 state on subsequent presentation of the stimulus, thus leading to reduced A1 activity. A2 state activity can occur due to the recent presentation of a stimulus (self-generated priming; route 2). Also, presentation of a stimulus leads to A2 state activation of elements of other stimuli with which it is associated (retrieval-generated priming; route 4).

Wagner's (1981) sometimes opponent process theory assumes that each stimulus representation consists of a set of stimulus elements. These stimulus elements can reside in any one of three different memory or activity states: a primary activity state (A1), a secondary activity state (A2) or an inactive state (I), although elements can only occupy one state at any one time, and only certain transitions between certain states are possible (see Fig. 4).

In the absence of the stimulus, the elements that represent that stimulus are in the inactive state (I). The presentation of a novel or surprising stimulus probabilistically activates a subset of the stimulus’ representational elements into the primary activity state referred to as A1 (via route 1). From here, the elements then rapidly decay into an A2 state, a process referred to as self-generated priming (route 2), before then gradually becoming inactive again via route 3 (i.e. returning to the I state). Elements can also be activated by presentation of cues that are associated with the target stimulus (i.e. by associative, retrieval-generated priming). Elements that are associatively activated enter the A2 state directly (via route 4). According to Wagner's SOP model, elements in the A1 state are able to generate strong responding, whereas elements in A2 are less able to do so. Also, excitatory associations (i.e. the formation of long-term memories) are formed between elements that are concurrently active in the A1 state.

Wagner (1981) proposed that short-term habituation reflects the accumulation of stimulus elements in the A2 state with each exposure to the stimulus (self-generated priming). When the stimulus is subsequently presented again, those elements in A2 cannot return to the A1 state. This results in a reduction in the proportion of elements that are available for activation in the A1 state. That, in turn, reduces the amount of unconditioned responding (e.g. exploration) elicited by the stimulus. In contrast, long-term habituation is thought to arise because the stimulus becomes associated with its experimental context. The presentation of the testing context (e.g. the Start arm) primes the memory of the increasingly familiar Sample arm. That is, the context primes activation of the stimulus elements directly into the A2 state (referred to as associative or retrieval-generated priming). This also reduces the proportion of elements available for activation into the A1 state on subsequent presentation of the stimulus and thus the amount of responding that it generates. Whereas self-generated priming is dependent on the recency of a stimulus presentation, retrieval-generated priming is dependent on the strength of the prior association formed between the context and the target stimulus.

In view of the preserved ability of *GluA1*^−/−^ mice to form associations involving spatial stimuli on reference memory tasks (Reisel et al., 2002; Schmitt et al., 2003; Zamanillo et al., 1999), we have suggested that long-term memory is preserved in these mice. In contrast, the impairments in spatial working memory performance suggest that short-term, non-associative memory processes may be disrupted in *GluA1*^−/−^ mice. If this is the case then *GluA1*^−/−^ mice should show normal long-term habituation, but impaired short-term habituation.

## 10 GluA1 knockout mice exhibit superior long-term spatial memory

**Fig. 5:**
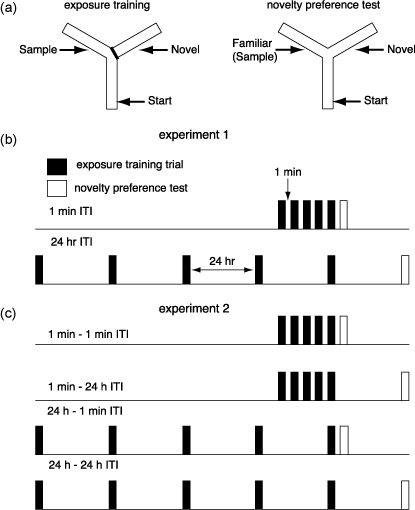
The design of Experiments 1 and 2 in Sanderson et al. (2009). (a) During exposure training mice were allowed to explore the Start arm and the Sample arm for five 2-min trials. Access to the Novel arm was blocked. During the novelty preference test mice were allowed to explore the two familiar arms (Start and Sample) and the previously unvisited, Novel arm for a period of 2 min. (b) In Experiment 1 the interval between exposure trials (represented by the black bars) and also the interval prior to the novelty preference test (represented by the white bars) was either 1 min (1 min ITI) or 24 h (24 h ITI). (c) In Experiment 2, two groups of mice from each genotype received exposure training with a 1 min interval between trials and two further groups from each genotype received exposure training with a 24 h interval between trials. One group from each training condition received the novelty preference 1 min after the last training trial. The other group received the test 24 h after the last training trial.

In a recent study, we tested the effects of *GluA1* knockout on short-term and long-term habituation (Sanderson et al., 2009). Spatial habituation was again assessed using the novelty preference Y-maze test which involved a simultaneous choice between a familiar, previously exposed, spatial location and a novel location (see Fig. 5a). However, the paradigm was adapted to include repeated training trials prior to a single preference test. Short- and long-term habituation to spatial cues were assessed by manipulating (i) the length of the interval between the series of exposure training trials and (ii) the interval prior to the test of spatial memory.

In the first experiment, mice received five 2-min exposure training trials to two arms of the Y-maze (the Start and Sample arms; see Fig. 5b), prior to a novelty preference test during which they could choose between the two familiar arms and a Novel (unexposed) arm (Sanderson et al., 2009). In one condition, there was a 1 min inter-trial interval (ITI) between each of the training trials, and a 1 min interval between the last training trial and the test trial. In the other condition, the gap between each of the training trials, and between the last training trial and the preference test was 24 h (see Fig. 5b). When the short, 1 min ITI is used, performance should be maximally influenced by short-term memory processes. However, when the long, 24 h ITI is used, performance should reflect long-term memory.

**Fig. 6:**
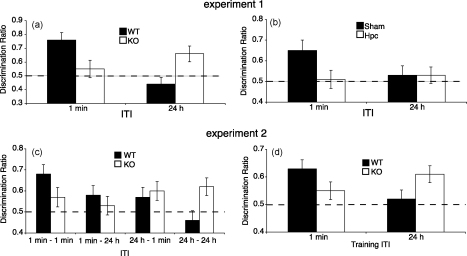
*GluA1* knockout impairs short-term spatial novelty preference, but enhances long-term novelty preference. The preference for the Novel arm over the Familiar (Sample) arm is shown as a discrimination ratio (Novel/[Novel + Familiar]). Scores greater than 0.5 indicate a novelty preference. The dashed lines indicate chance performance. Errors bars indicate ±S.E.M. (a) In Experiment 1 (see Fig. 5b) *GluA1*^−/−^ mice (KO) were enhanced in the long, 24 h ITI condition relative to wild-type mice (WT), but impaired in the short, 1 min ITI condition. (b) Hippocampal lesioned mice (Hpc) failed to show a significant novelty preference in either condition. (c) In Experiment 2 (see Fig. 5c) *GluA1*^−/−^ mice were enhanced when the training trials were separated by 24 h. There was no significant interaction between genotype and the test interval. (d) The results of Experiment 2 collapsed across the short, 1 min and long, 24 h test intervals to show the independent effects of the training ITI in wild-type and *GluA1*^−/−^ mice. Data reproduced from Sanderson et al. (2009).

During the novelty preference test wild-type mice showed a strong preference to explore the Novel arm in the 1 min ITI condition, whereas *GluA1*^−/−^ mice did not show this preference. However, in the 24 h ITI condition, *GluA1*^−/−^ mice showed a stronger novelty preference than the controls (Fig. 6a). Thus, this pattern of results was consistent with our view that long-term spatial memory was intact in *GluA1*^−/−^ mice, and that habituation supported by short-term memory processes is impaired.

Nevertheless, there are two possible explanations for the interaction between the effects of *GluA1* deletion and the ITI in this experiment. *GluA1* deletion could differentially affect acquisition of learning that occurs in the massed (1 min ITI) and spaced (24 h ITI) conditions. Alternatively, *GluA1* deletion may differentially influence the expression of memory either across short (1 min), or long (24 h) intervals. To test these two hypotheses, both the interval between exposure training trials and the interval prior to the novelty preference test were manipulated in a between-subjects factorial design (see Fig. 5c).

Mice received exposure training with trials separated by either a 1 min or a 24 h ITI as in the previous experiment (Sanderson et al., 2009). After exposure training, half the mice from each ITI condition then received the novelty preference test after 1 min, and the remaining mice received the test after 24 h. In agreement with Experiment 1, *GluA1*^−/−^ mice showed a stronger novelty preference than controls when the training ITI was 24 h (Fig. 6c and d). However, when the training ITI was short (1 min) the knockout mice showed a weaker novelty preference than controls. This pattern was not influenced by the interval between the last training trial and the novelty test. Thus, the enhanced spatial recognition memory in the *GluA1*^−/−^ mice was the result of the interval between the training trials and not the result of the interval prior to the novelty preference test, thus demonstrating enhanced learning and not simply enhanced performance or expression of memory.

Importantly, both short-term and long-term spatial habituation were disrupted by hippocampal lesions (Sanderson et al., 2009). In the novelty preference test, lesioned mice failed to show a novelty preference with either short or long ITIs (Fig. 6b). Therefore, the enhanced long-term spatial memory in *GluA1*^−/−^ mice appears to reflect memory supported by the hippocampus. This facilitation of long-term spatial memory in *GluA1*^−/−^ mice is an important result for a number of reasons. The most striking of these is that it actually demonstrates enhanced spatial memory in animals lacking a form of hippocampal synaptic plasticity.

Furthermore, several accounts of the short-term spatial memory deficit in *GluA1*^−/−^ mice can be ruled out with this data set. First, these mice are not only able to discriminate between different spatial locations (see also Reisel et al., 2002; Schmitt et al., 2003; Zamanillo et al., 1999), but they can do so on the basis of novelty preference. Second, any simple non-specific effects of *GluA1* deletion on locomotor activity or motivational states cannot explain these time-dependent effects on learning. No possible confound could cause the diametrically opposite effect on short-term and long-term learning using the same measures of performance. Importantly, two different performance measures (time in arms and number of arm entries) both demonstrated the same pattern of results (see Sanderson et al., 2009).

The facilitation observed with long interval training also argues against a simple, partial degradation of hippocampal function in *GluA1*^−/−^ mice. It has been suggested that the pattern of impaired short-term spatial memory, but spared long-term spatial memory observed in these mice could reflect a non-specific, but incomplete, disruption of hippocampal function, with short-term memory (or spatial working memory) performance simply being the more sensitive measure. The demonstration of facilitated long-term spatial memory in *GluA1*^−/−^ mice argues strongly against this possibility and implies that GluA1 contributes to an interaction between short-term and long-term memory systems.

Furthermore, these results do not fit with a simple trace decay interpretation of memory. They argue against a model whereby short-term memories are serially converted into long-term memories. Instead, these data argue for two dissociable memory processes. Indeed the fact that *GluA1* deletion was able both to impair short-term spatial memory, and enhance long-term memory suggests that these forms of memory depend on separate psychological processes that can, under some circumstances, compete with one another. Thus, these results also do not support a single process account of habituation (Horn, 1967; Mackintosh, 1987), but are consistent with a dual-process memory model (Wagner, 1976, 1981).

## 11 A dual-process memory model

As described in Section 9, Wagner (1976, 1981) suggested that there are independent short-term and long-term memory processes that can both contribute to habituation. He suggested that short-term habituation reflects self-generated priming of the memory of a recently presented stimulus. In contrast, long-term habituation is thought to reflect associative, retrieval-generated priming of the memory for a stimulus. Long-term habituation is thus based on associations formed between the target stimulus and contextual cues, and therefore, the extent of the habituation is dependent on the strength of these associations. For example, in the Y-maze spatial novelty preference task, animals may learn to associate the Start arm, or indeed other cues defining the experimental context, with the Sample arm, during the training trials. Thus, it may be that the spatial cues experienced in the Start arm prime the memory of the Sample arm, resulting in habituation to that arm and hence a preference to explore the Novel arm during the test trial.

The pattern of impaired short-term habituation and enhanced long-term habituation in *GluA1*^−/−^ mice suggests that short-term and long-term processes compete with one another. This idea can be accommodated by Wagner's (1981) theory. A feature of associative learning is that increments in long-term learning are greater when the occurrences of the stimuli are surprising (Rescorla & Wagner, 1972; Wagner, 1981). Of course, habituation renders stimuli less surprising. Therefore, a short-term memory of a stimulus can retard subsequent associative learning by rendering the occurrence of the stimulus unsurprising, and thus reducing the levels of attention that are subsequently paid to that stimulus (Wagner, 1981). For example, Sunsay, Stetson, and Bouton (2004) have shown that the recent presentation of a stimulus (CS1) impairs the ability of that stimulus to enter into associations with other stimuli when subsequently paired (CS1-US). This retardation in conditioning was not seen if a different stimulus (CS2) preceded the CS1-US pairing. The impairment in conditioning is due to the fact that a stimulus-specific short-term memory of the target (CS1) reduces subsequent processing of that target cue and thus disrupts long-term, associative learning.

The fact the GluA1 deletion enhanced long-term spatial memory whilst impairing short-term spatial memory suggests that the short-term memory processes may reduce the extent or speed with which long-term memories of the environment are formed by control mice in the 24 h ITI condition. This is consistent with the theoretical view proposed by Wagner (1981) in which short-term memory processes can influence the encoding of events in long-term memory. For example, novel cues enter the A1 state in Wagner's model, which can be equated to the focus of attention, where associations are formed between co-active stimuli. Elements in the A1 state decay over time into the A2 state, where they are less effectively processed. Elements in A2 are not available to enter the A1 state where associations can be formed. Eventually these elements return to the inactive or long-term memory store. Thus, in wild-type mice, elements in the short-term memory A2 state would be expected to build up within a trial. If long-term memory is dependent on the formation of associations between concurrently active stimuli, then the build up of short-term memory within a trial will limit the amount of long-term learning that can occur. Thus, within a trial the presence of elements in the A2 short-term memory state will reduce the opportunity for these representational elements to enter into associations (and thus to be stored in long-term memory).

Based on Wagner's analysis of habituation, one interpretation of our data is that *GluA1* deletion influences the transfer into, or maintenance of representational elements in, the short-term memory A2 state. If this were the case then these mice should show impaired short-term habituation to spatial stimuli within a trial. Furthermore, this could increase the opportunity for further processing of cues (in the A1 memory state), thus enhancing associative long-term memory formation. This hypothesis is not without precedent. The idea that the formation of short-term memory within a trial may impair long-term associative learning is supported by the finding that conditioning is greater with a conditioned stimulus (CS) of intermediate length duration, compared to a longer CS duration (Smith, 1968). This is because a longer CS may undergo greater short-term habituation (i.e. more of its representational elements are in the A2 state) by the time the unconditioned stimulus (US) is presented. This suggests that *GluA1* deletion may facilitate long-term learning by reducing the negative within-trial effects of short-term memory.

According to our analysis, *GluA1* deletion may impair short-term habituation and enhance long-term habituation because it affects the rate of transfer between these different activity states. *GluA1* deletion may influence these processes in a number of ways. For example, *GluA*1 deletion may reduce the rate at which elements transfer from A1 to A2, or reduce the capacity of A2, or alter the extent to which elements remain active in the A2 state. All of these possibilities could result in impaired short-term habituation, because there would be fewer elements in the A2 state. In addition, because stimulus elements would therefore remain in the A1 state for longer, or be more frequently activated into the A1 state, then there would be an increased opportunity for the formation of long-term associations. This increase in associative learning could then subsequently lead to greater retrieval-generated priming and hence stronger long-term habituation.

**Fig. 7:**
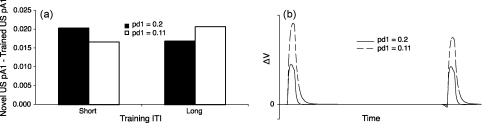
Simulations of Wagner's SOP model (1981). To simulate the effect of *GluA1* deletion on spatial novelty preference, calculations were performed to determine the difference in the memory states following two exposure training trials followed by a novelty test. It is assumed that during an exposure trial there are elements of a stimulus that are active throughout the trial (e.g. a CS) that can form an association, and thus predict the occurrence of elements of another stimulus that are only active towards the end of the trial (e.g. a US). This may, for example, describe the possible associative learning between spatial stimuli experienced in the Start arm and spatial stimuli experienced in the Sample arm. The CS is a 10-moment stimulus and the US is a 5 moment stimulus that co-terminated. The short and long training ITI were a length of 5 and 100 moments respectively. The novelty preference test consisted of 6 moments of the CS and 1 moment of the trained US and a novel US that co-terminated. For all stimuli the intensity parameter was 0.2. For normal wild-type mice the decay parameters for A1 (pd1) and A2 (pd2) were 0.2 and 0.04 respectively. For *GluA1*^−/−^ mice the value of pd1 was reduced to 0.11. The excitatory learning rate parameter was 0.07 and the inhibitory learning rate parameter was 0.014 (for other details see Sanderson et al., 2009). Simulations were carried out for training with a short and a long ITI and with testing after a short or long interval for both conditions. The results are subsequently collapsed across testing conditions so as to show the independent effects of the training ITI. (a) The difference in A1 activity between a novel US and a trained US. A positive value indicates greater relative novel US A1 activity. When pd1 = 0.2, a short training ITI causes a greater relative novel US A1 activity than a long training ITI. However, when pd1 = 0.11, a long training ITI causes a greater relative novel US A1 activity than a short training ITI. (b) The momentary change in net associative strength (Δ*V*) over two exposure training trials. A slower A1 to A2 decay rate (pd1 = 0.11) causes greater increments in associative strength than a faster decay rate (pd1 = 0.2) by increasing the concurrent CS and US A1 state activity.

These predictions were tested using mathematical approximations of Wagner's SOP model. During an exposure trial it is possible that associative learning can occur between spatial stimuli experienced in the Start arm and spatial stimuli experienced in the Sample arm. This requires elements of these spatial stimuli to be concurrently active in the A1 state. The amount of A2 activation during the novelty preference test (and hence the extent of any novelty preference) is determined by the extent of both self-generated and associative, retrieval-generated priming (for further details of the simulation see Fig. 7). Thus, the amounts of A1 activation caused by a familiar cue after exposure training were simulated and compared to the activation in A1 caused by a novel cue, thus reproducing the spatial novelty preference Y-maze test. Greater A1 activation produced by the novel stimulus compared to that caused by the familiar stimulus indicates a novelty preference.

For controls, parameters were chosen that resulted in a strong novelty preference after short ITI training and a weak novelty preference after long ITI training. Thus, the rate of transfer from A1 to A2 states resulted in a rapid accumulation of elements in A2, resulting in short-term habituation. This, however, reduced the amount of A1 activation such that there was little associative learning (e.g. between the Start and Sample arms), and consequently only weak long-term habituation. The effects of GluA1 deletion were simulated by reducing the rate of transfer from A1 to A2. It was found that a 16.5–56.5% reduction in this transfer rate led to a reduction in the novelty preference in the short ITI novelty preference test, but, at the same time, produced a greater novelty preference in the long ITI condition (see Fig. 7a). The reduced rate of A1–A2 transfer resulted in a reduction in self-generated A2 priming, thus reducing short-term habituation, but increased the amount of A1 activation such that there was an increase in excitatory, associative learning and thus greater long-term habituation (see Fig. 7b).

Thus, simulations of Wagner's SOP model demonstrate that a reduction in the rate of transfer of stimulus elements from active processing (the A1 state) to the A2 state can cause greater long-term memory whilst reducing short-term memory. So how might these psychological processes map onto neuronal networks within the hippocampal formation or elsewhere, and what is the role of synaptic plasticity in these processes?

## 12 The role of the hippocampus in attentional processes

The majority of models of hippocampal function suggest that memories are formed in the hippocampus and are stored there, at least temporarily. In the case of the cognitive map hypothesis, it is suggested that the spatial memory representation is maintained there permanently (O’Keefe & Nadel, 1978). However, others have suggested that the memory representations may be formed and maintained in the cortex, with the hippocampus playing a very different role (Gray, 1982; Gray & McNaughton, 2000; Honey & Good, 2000). For example, it has been suggested that the anatomy of the hippocampus is ideally suited to act as a comparator, for detecting novelty and mismatch (associative novelty). This involves comparing the present state of the world with what is expected on the basis of associatively retrieved information from memory (Gray, 1982; Gray & McNaughton, 2000; Vinogradova, 1975, 2001). Gray (1982) suggested that, following the detection of novel or unexpected events, an important function of the hippocampus is to increase levels of arousal and attentional processing, and thus increase investigation. If the information retrieved from memory does not match the incoming sensory input (e.g. under conditions when a novel stimulus is presented), then responding in the form of exploration of the stimulus is maintained. Conversely, habituation occurs when the memory of a stimulus matches incoming sensory information. Furthermore, Gray also suggested that the hippocampus may play an important role in modulating the processing of stimuli by reducing the attentional intensity or perceived salience of recently presented, familiar stimuli.

Indeed, the hippocampus may play a very important role in habituation processes, not only for spatial, but also non-spatial stimuli (Marshall, McGregor, Good, & Honey, 2004). A recent study by Honey, Marshall, McGregor, Futter, and Good (2007) provided intriguing evidence in support of this idea in the spatial domain. Honey et al. (2007) showed that rats with hippocampal lesions were more likely to re-visit recently visited places within an open field compared to control rats (see also Marshall et al., 2004). Furthermore, lesioned animals showed greater exploration of a context that they had just recently explored than a less recently visited context, an effect that, again, was not evident in controls. This short-term sensitisation phenomenon (increased investigation of a recently experienced stimulus) suggests that, surprisingly, a spatial representation does in fact exist in rats with hippocampal lesions. Otherwise, it would not be possible for those animals to show any form of spatial preference. Instead, these results argue for a role for the hippocampus in modulating the attentional intensity or perceived salience of stimulus representations that reside elsewhere, presumably in the cortex.

Indeed, the anatomy of the hippocampal formation, and the close interaction between the hippocampus and the cortex, seem ideally suited to support such an information processing dynamic. The uni-directional loop through the hippocampal formation may be particularly well suited for priming of spatial memories. Importantly, the return projections from CA1 (and subiculum) to ERC send their axons back to the same region of ERC from which they received their inputs, thus completing a very specific loop within the hippocampal formation (Naber, Lopes da Silva, & Witter, 2001). This may provide a mechanism, at least globally, by which the neural circuitry of the hippocampal formation might allow the activation of a specific spatial stimulus representation to modify its own attentional intensity for subsequent stimulus presentations (e.g. for repetition suppression), a property that could underlie self-generated priming. Furthermore, recent computational models have emphasised the importance of dentate gyrus-CA3 connections for rapidly representing novel memory traces on each presentation of a spatial stimulus, a key requirement for self-generated priming (Treves, Tashiro, Witter, & Moser, 2008).

Thus, an interplay between the hippocampus and stimulus representations in the cortex may represent a mechanism by which the attentional intensity or perceived salience of a stimulus may be reduced, thus leading to habituation. Of course, this would require plastic changes at appropriate synapses within the network. The idea that synaptic plasticity within the hippocampal formation may be a mechanism for modulating the attentional processing of stimuli has been suggested previously (Shors & Matzel, 1997). We now suggest that GluA1-dependent plastic changes may underlie stimulus-specific, short-term reductions in the attentional processing of stimuli.

## 13 The role of synaptic plasticity in Wagner's model

A key starting point is Wagner's postulate that the stimulus is made up of a number of elements. It seems reasonable to equate Wagner's elements with neurons in the cortex, such that the elements that make up a stimulus correspond to the collection of neurons that fire in response to the presentation of a given stimulus. Under normal conditions, in the absence of the stimulus, these neurons remain inactive in the cortex (thus, analogous to Wagner's inactive state, I). Elements in the A1 state are better able to generate responding than elements in the A2 state. Furthermore, only elements in A1 are able to form excitatory associations. Thus, A1 activity may correspond to the firing of the neuron and the generation of an action potential, which would of course be consistent with Hebb's postulate that neurons that fire together wire together (Hebb, 1949). Elements in A2 *are* able to generate some responding, but this is much weaker, and they are not able to form excitatory associations. Thus, the A2 state may resemble a kind of refractory state, and if we think of Wagner's elements in terms of neurons, then the A2 state may represent a condition in which the neurons that represent the stimulus become less excitable. If so, then short-term habituation may involve a reduction in the excitability of the neuronal network that represents a given spatial location.

Consistent with this hypothesis, there is evidence from human fMRI studies that neuronal activity is reduced when stimuli are repeated, compared to when novel stimuli are presented. The phenomenon of repetition suppression, when a recently presented, familiar stimulus is presented again, is associated with a reduction in the BOLD signal (Brown & Aggleton, 2001; Grill-Spector, Henson, & Martin, 2006; Henson et al., 2003; Kumaran & Maguire, 2006; Ranganath & Rainer, 2003). Indeed, it has been suggested that repetition suppression may be mediated by the tuning or modulation of neuronal representations such that familiar stimuli will activate fewer neurons and thus evoke less neuronal activity (Kumaran & Maguire, 2009). Consistent with this suggestion, there is considerable evidence from single cell recordings in animals that repetition suppression is associated with a decrease in neuronal firing, at least in some brain regions (e.g. Brown & Aggleton, 2001; Brown, Wilson, & Riches, 1987; Grill-Spector et al., 2006).

If short-term habituation is associated with a reduction in neuronal activity, then it seems unlikely that LTP or STP, which increase the excitability of neurons, would be the appropriate neurobiological substrate. It has previously been suggested that long-term depression (LTD), rather than LTP, may provide the mechanism that underlies familiarity discrimination for visual stimuli such as objects (Brown & Bashir, 2002; Griffiths et al., 2008; Warburton et al., 2003). It is possible that a similar LTD mechanism (or a short-term depression or depotentiation) could be responsible for short-term habituation to spatial stimuli. For example, NMDA receptor-dependent LTD depends on the internalization of AMPA receptors. It has been proposed that this depends more on the GluA2 subunit, but it has also been suggested that phosphatase activity, leading to the dephosphorylation of GluA1 at key amino acid residues, can result in the removal of GluA1-containing AMPA receptors from synapses (see Kessels & Malinow, 2009). Indeed, genetically modified mice that express GluA1 subunits lacking certain key phosphorylation sites (S831A, S845A), fail to exhibit NMDA receptor-dependent LTD (Hu, Huang, Yang, & Xia, 2007; Lee et al., 2003). Thus, NMDA receptor-dependent LTD requires GluA1 dephosphorylation. However, the effect of a constitutive, deletion of the GluA1 subunit on LTD expression still remains to be established.

An alternative possibility is that in normal animals repetition suppression is accompanied by an increase in the activity of GABA-ergic inhibitory interneurons, resulting in a reduction in the excitability of the principal cells. This could involve GluA1-dependent synaptic plasticity (LTP or STP) onto inhibitory interneurons such that *GluA1* deletion prevents any increase in interneuron activity levels, and thus prevents repetition suppression. Consistent with this possibility, deletion of GluA1 exclusively from a subset of parvalbumin-positive interneurons also produced a pronounced spatial working memory deficit in the presence of preserved spatial reference memory performance (Fuchs et al., 2007).

Whatever the mechanism that leads to neurons entering into a refractory state, it would appear that separate mechanisms must exist for self-generated, and associative, retrieval generated, priming of memories into the A2 state. Our studies with *GluA1*^−/−^ mice suggest separate GluA1-dependent and GluA1-independent mechanisms respectively are involved in these distinct aspects of information processing. The challenge now is to identify which plasticity mechanism (or mechanisms) supports these behaviours, and at what set (or sets) of synapses these plastic changes might occur.

## 14 Frontal cortex and short-term memory

So far this article has been concerned exclusively with memory in the medial temporal lobe and its rodent equivalent. However, the term working memory is also used in conjunction with a separate short-term memory system associated with the frontal lobe. Indeed, the short-term/working memory terminology constitutes a potential problem, and is a frequent source of misunderstanding. In particular, different researchers have used the phrase ‘working memory’ to reflect different behaviours, associated with either the hippocampus or the frontal lobe (e.g. Baddeley & Hitch, 1974; Goldman-Rakic, 1987; Olton et al., 1979). A better understanding of the psychological processes involved in these behaviours may help to delineate hippocampal and frontal contributions to short-term memory. In particular, the suggestion that spatial working memory performance on win-shift maze tasks reflects a hippocampus-dependent, short-term habituation process, through which animals gauge the relative level of familiarity of the spatial stimuli, may help to clarify this situation. This is clearly a very different psychological process from that recruited during human tasks such as the N-back task or backward digit span tasks which reliably activate frontal cortex (Hoshi et al., 2000; Jansma, Ramsey, Coppola, & Kahn, 2000; McMillan, Laird, Witt, & Meyerand, 2007; Morgen et al., 2006; Rama et al., 2001), and which are affected by frontal damage (Morgen et al., 2006; Ptak & Schnider, 2004; Tsuchida & Fellows, 2009). Nevertheless, before returning to the psychological differences between frontal and hippocampal working memory, we will first briefly review the effects of frontal damage on win-shift maze tasks.

Although older studies using traditional, non-fibre-sparing lesions (e.g. aspiration lesions) of the prefrontal cortex often resulted in impaired spatial alternation (for a review see Kolb, 1984), more recent studies employing cytotoxic, fibre sparing techniques have found that lesions of medial prefrontal cortex (mPFC) by no means robustly affect non-matching-to-place performance in rodents (Aggleton, Neave, Nagle, & Sahgal, 1995; Deacon, Penny, & Rawlins, 2003). Furthermore, where deficits do exist they are generally mild and transient in nature (Delatour & Gisquet-Verrier, 2000; Dias & Aggleton, 2000; Kellendonk et al., 2006; Sanchez-Santed, de Bruin, Heinsbroek, & Verwer, 1997; Shaw & Aggleton, 1993; Walton, Bannerman, Alterescu, & Rushworth, 2003), or they involve the introduction of an additional delay period between choices (Di Pietro, Black, Green-Jordan, Eichenbaum, & Kantak, 2004; Floresco, Seamans, & Phillips, 1997; Seamans, Floresco, & Phillips, 1995; Taylor, Latimer, & Winn, 2003). Indeed, rodents with mPFC lesions are perfectly capable of high levels of spatial, non-match to place performance (e.g. Touzani, Puthanveettil, & Kandel, 2007), and even at longer delays (Gisquet-Verrier & Delatour, 2006). Gisquet-Verrier and Delatour suggested that the mPFC “is not directly involved in the short-term maintenance of specific information, but is implicated when changes, such as the sudden introduction of a delay or exposure to unexpected interfering events, alter the initial situation” (Gisquet-Verrier & Delatour, 2006, p. 585; see also Touzani et al., 2007). This contrasts markedly with the effects of hippocampal lesions.

Along similar lines, studies have shown that rats with damage, specifically in the prelimbic/infralimbic (PL/IL) region of the medial prefrontal cortex, are slower to switch from non-matching, win-shift behaviour to adopt a matching-to-place rule (Walton et al., 2003). Rats were trained on a matching-to-place version of the T-maze task in which they were required to visit the same arm on the choice run as on the sample run to obtain a reward. Because animals have a natural propensity to alternate, they start off at well below chance levels of performance (i.e. below 50% correct choices). Performance on the early training blocks therefore provides an assessment of alternation behaviour. Just like the sham operated group, rats with either anterior cingulate cortex (ACC) lesions or with PL/IL lesions displayed very low levels of performance on the matching-to-place task during these early blocks (i.e. they were well below chance levels), consistent with preserved alternation in these animals. Indeed, during the early stages of the experiment the levels of alternation were indistinguishable in the three groups. However, PL/IL lesioned animals were then slower in switching from non-matching/alternation behaviour to applying a matching rule (see also Dias & Aggleton, 2000). Thus, the PL/IL cortex does not appear to be important for the short-term memory of which arm was most recently visited, although it may be important for imposing a matching rule onto the rule-independent, natural propensity to alternate.

We have also recently examined the effects of orbitofrontal cortex lesions in rats on T-maze rewarded alternation performance (Mariano et al., 2009). Rewarded alternation was normal in OFC-lesioned rats, in marked contrast to the pronounced deficit in hippocampal lesioned animals in the same study. When the task was made harder by introducing a delay between the sample run and the choice run, the OFC-lesioned rats were still indistinguishable from the controls.

Therefore, spatial working memory as studied on win-shift maze tasks in rodents is dependent on the hippocampal formation, but may not depend on frontal lobe structures. That is not to say that frontal lesions never affect performance on these maze tasks, but rather that these effects, when observed, reflect other aspects of task performance and not the generation and/or maintenance of the short-term memory trace itself. This of course implies that there are categorical differences between working memory tasks that involve win-shift behaviour on mazes in rodents and commonly used tests of working memory in primates (e.g. N-back, digit span, delayed saccade in humans, delayed response in monkeys). Our description of win-shift maze performance in terms of short-term habituation clearly sets this form of working memory apart from a frontal, working memory system as usually studied in primates. Of course the ability to hold specific information on-line for subsequent recall or utilization during N-back or digit span tasks, for example, presumably reflects an ability to maintain an active stimulus representation in the absence of the stimulus itself. It would therefore be of great interest to investigate the effects of frontal lesions on aspects of Wagner's dual-process model.

## 15 Conclusions

To conclude, data from *GluA1*^−/−^ mice suggest that win-shift, spatial working memory performance reflects a non-associative, short-term habituation process in which animals choose more novel arms in preference to more familiar options. In contrast, spatial reference memory acquisition on tasks like the Morris water maze is likely to reflect associative, long-term memory processes. These results are potentially accommodated by Wagner's dual-process model of memory in which short and long-term mechanisms exist in parallel and, under certain circumstances, can compete with each other. *GluA1*^−/−^ mice lack a form of short-term memory for recently experienced spatial stimuli, but as a consequence, these stimuli remain surprising and so are better able to form long-term associations. We have recently shown that long-term spatial memory is enhanced in *GluA1*^−/−^ mice, despite impairments in hippocampal synaptic plasticity. Taken together, these results support a role for GluA1-containing AMPA receptors in short-term habituation, and are consistent with a role for the hippocampus in modulating the attentional intensity or perceived salience of spatial stimuli.

Finally, it is also worth noting that a role for the hippocampus, and for synaptic plasticity, in modulating attentional intensity and perceived stimulus salience may have important implications for certain psychiatric disorders, most notably schizophrenia. NMDA receptor hypofunction is widely thought to contribute to cognitive dysfunction in schizophrenia, and attentional deficits are a core symptom of this disease. Furthermore, it has been suggested that hallucinations and delusions may reflect the formation of inappropriate associations between stimuli, something that would not normally occur in healthy individuals (Frith, 1996; Gray, Feldon, Rawlins, Hemsley, & Smith, 1991; Hemsley, 1994; Kapur, 2003). In this respect, it is interesting that the enhanced long-term spatial recognition memory in the *GluA1*^−/−^ mice could be considered as an inappropriate association that did not form in the wild-type mice. Thus, an inability to reduce the attentional intensity of stimuli, as a result of deficits in synaptic plasticity in the hippocampal formation and/or elsewhere in cortex, may result in inappropriate associations being formed, which, in turn, may have serious consequences for the patient's perception of his or her external environment.
